# Thyroid cancer cells in space during the TEXUS-53 sounding rocket mission – The THYROID Project

**DOI:** 10.1038/s41598-018-28695-1

**Published:** 2018-07-09

**Authors:** Sascha Kopp, Marcus Krüger, Stefan Feldmann, Hergen Oltmann, Andreas Schütte, Burkhard Schmitz, Johann Bauer, Herbert Schulz, Kathrin Saar, Norbert Huebner, Markus Wehland, Mohamed Zakaria Nassef, Daniela Melnik, Stefan Meltendorf, Manfred Infanger, Daniela Grimm

**Affiliations:** 1 Clinic for Plastic, Aesthetic and Hand Surgery, Otto-von-Guericke-University Magdeburg, Leipziger Str. 44, D-39120 Magdeburg, Germany; 2 Airbus Defence and Space GmbH, Airbus-Allee 1, D-28199 Bremen, Germany; 3 Max-Planck Institute of Biochemistry, D-82152 Martinsried, Germany; 4 Cologne Center for Genomics, University of Cologne, D-50931 Cologne, Germany; 5 Max-Delbrück-Center for Molecular Medicine, D-13092 Berlin-Buch, Germany; 6 Experimental Pediatrics and Neonatology, University Hospital, Otto-von-Guericke University Magdeburg, Leipziger Str. 44, D-39120 Magdeburg, Germany; 7 Department of Biomedicine, Aarhus University, Wilhelm Meyers Allé 4, DK-8000 Aarhus C, Denmark; 8 Gravitational Biology and Translational Regenerative Medicine, Faculty of Medicine and Mechanical Engineering, Otto-von-Guericke-University Magdeburg, D-39120 Magdeburg, Germany

## Abstract

Human follicular thyroid cancer cells (FTC-133) were sent to space via a sounding rocket during the TEXUS-53 mission to determine the impact of short-term microgravity on these cells. To enable cell culture and fixation in real microgravity, an automated experiment container (EC) was constructed. In order to ensure safe cell culture, cell-chambers consisting of polycarbonate (PC) material were used. They were highly biocompatible as proved by measuring cell survival using Annexin V flow cytometry. In the follow-up experiment, FTC-133 cells were sent to space via a sounding rocket and were fixed before and after the microgravity (µ*g*) phase with RNA*later*. In addition, cells were tested for reactions on hypergravity (hyper-*g*) as much as 18 *g* to determine whether worst case acceleration during launch can have an influence on the cells. We investigated genes belonging to biological processes such as cytoskeleton, cell adhesion, tumor growth, angiogenesis and apoptosis. Pathway analyses revealed central functions of *VEGFA* and *EGF*. *EGF* upregulates aspartate beta-hydroxylase (*ASPH*) which is influencing *CASP3*. Hyper-*g* induced a significant up-regulation of *TUBB1*, *VIM*, *RDX*, *CAV1*, *VEGFA* and *BCL2*. FTC-133 cells grown in an automated EC exposed to µ*g* revealed moderate gene expression changes indicating their survival in orbit.

## Introduction

Thyroid cancer is the most common malignancy of the endocrine system. The incidence of this cancer type continues to rise steadily worldwide^1^. According to GLOBOCAN, 298,102 new cases were diagnosed in the world population and 39,769 people died from thyroid cancer in 2012. Thyroid cancer comprises a group of tumors with different features^2^. Whereas differentiated thyroid cancer (DTC; papillary or follicular) types are well treatable and usually curable, poorly differentiated tumors are aggressive, metastasize early and have a much poorer prognosis^3^. Unfortunately, recurrent DTC can become less-differentiated, lack iodine uptake capability and is radioiodine refractory. Patients with this cancer type have a remarkably reduced survivability and treatment options for DTC are extremely limited. Therefore, new ideas in terms of drug development are needed to fill this treatment gap^3,4^.

Alterations of gravity have been shown to remarkably influence growth and biological processes of malignant cancer cells^5–10^. Thus, altered gravity experiments became a promising method to improve our understanding of thyroid cancer biology, and may be useful to detect interesting target proteins for future cancer treatment.

Dedifferentiated thyroid cancer cells of the cell line FTC-133 had already been exposed to short-term microgravity (22 seconds) during parabolic flights and to long-term microgravity attained during the SIMBOX/Shenzhou-8 mission and the CELLBOX-1 mission^11–14^.

While parabolic flights offer an accumulation of 31 parabolas each with 22 sec of microgravity (µ*g*) and 40 sec of 1.8 *g*, a space mission offers up to a 14-day or longer phase of microgravity^12,15–19^.

FTC-133 cells were fixed with RNA*later* and paraformaldehyde respectively during these missions, after the µ*g*-phases and were then analyzed post-flight in the respective laboratories. While short-term µ*g*-exposure resulted in gene expression changes which could be interpreted as an increase in tumor cell malignancy, cultivation of the same cell line in space for 10 days resulted in a gene expression profile hinting to a highly decreased malignancy in addition to three-dimensional (3D) growth^11,12^.

**Figure 1:**
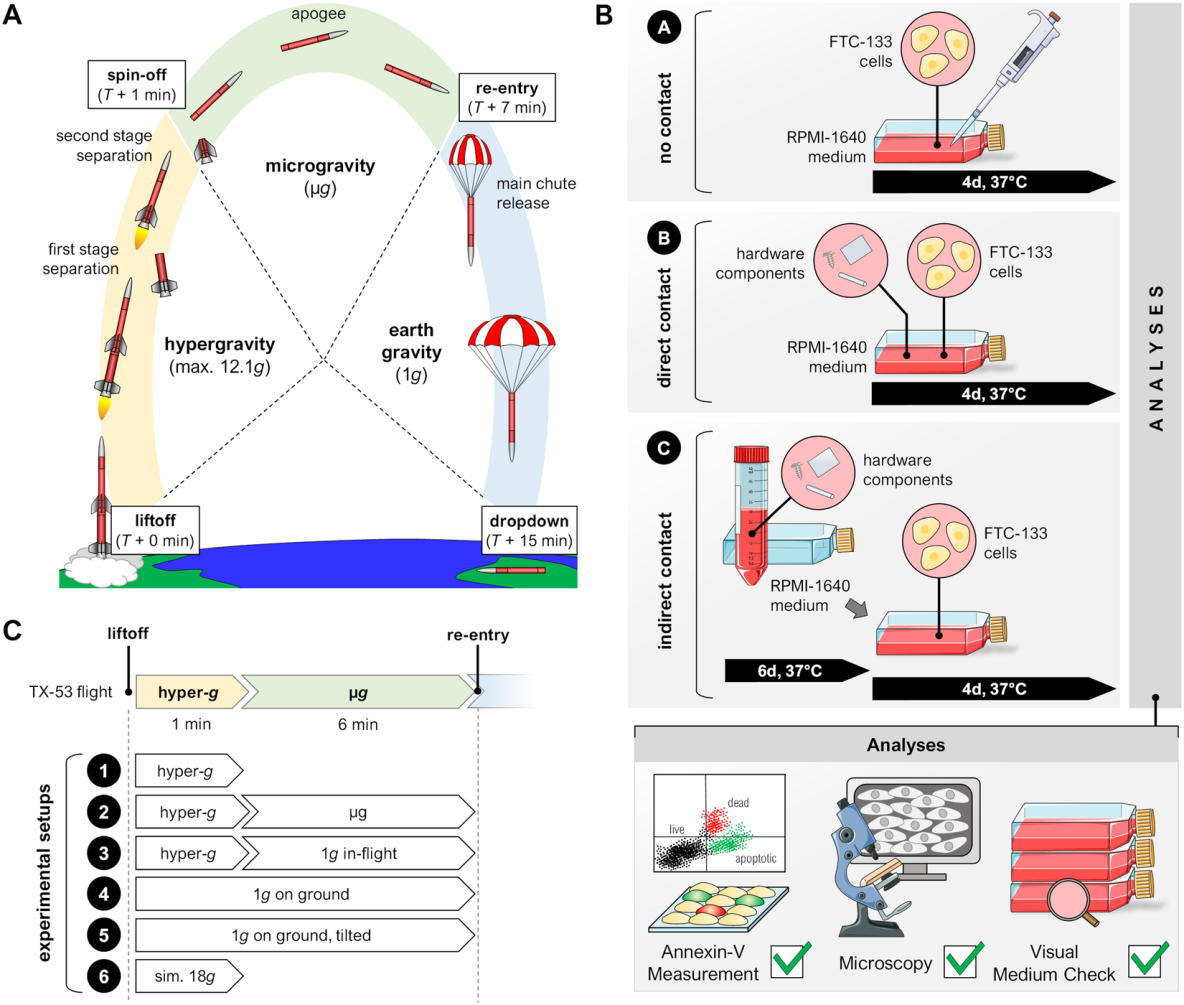
Experimental setup of the study for a rocket mission. (**A**) rocket flight; (**B**) biocompatibility experiments; (**C**) different experiment groups. Parts of the figure were drawn by using pictures from Servier Medical Art. Servier Medical Art by Servier is licensed under a Creative Commons Attribution 3.0 Unported License (https://creativecommons.org/licenses/by/3.0/).

Furthermore, FTC-133 cells expressing Lifeact-GFP marker protein were investigated during a sounding rocket mission using a compact fluorescence microscope (FLUMIAS) for fast live-cell imaging under real microgravity, revealing rapid cytoskeletal changes^18^. To examine the underlying mechanisms of these changes we exposed FTC-133 cells to 6 minutes of µ*g* during a sounding rocket mission (Fig. 1).

Based on data from these experiments we participated in the sounding rocket mission with the acronym TEXUS 53 (*Technologische Experimente unter Schwerelosigkeit*) in January 2016. The launch center was the Esrange Space Center in Kiruna, Sweden, which is operated by the Swedish Space Corporation. Requirements for the rocket flight were manifold. Firstly, the cell culture chambers to be used had to be adaptable to the changed conditions of a use in space, but also be biocompatible with the human thyroid cancer cells and the liquids utilized. Secondly, the cell chambers needed a sufficient growth area in order to harvest sufficient material for further analyses together with a significant number of replicates. In addition, an onboard 1*g*-centrifuge was necessary to discriminate between µ*g*-effects on the cells, launch and vibrations effects. Finally, the cell chambers had to be flushed with the fixative RNA*later* via a remote controlled operation at the time points of interest.

The major aim of this study was therefore to develop the experimental hardware for adherent cells, suitable to be operated during a rocket flight in real microgravity. Consequently, a cell container combined with a module allowing automatic fluid exchange and entrainment of replacement culture medium and fixatives was developed. This manuscript is comprised of a chronological description of this experiment and the post-flight evaluation of the resulting samples. It is completed by the investigation of the cells’ gene expression in real microgravity (r-µ*g*) and hyper-*g*.

## Results

To examine the mechanisms of gene expression changes of FTC-133 cells during the 6-min lasting µ*g*-phase of a sounding rocket mission (Fig. 1A), it was necessary to recover RNA-stabilized cells after the mission. Therefore, the company Airbus Defence and Space designed, assembled and operated a hardware suitable to save the treated cells until return and from material which passed the biocompatibility test (Fig. 1B). The hardware is thermo-controlled to 37 °C and easy access to the hardware in the rocket is possible by separate openings in the outer structure so that EUs could be installed at the latest possible time before launch. The experimental setup is given in detail in Fig. 1C.

### Biocompatibility Test

FTC-133 thyroid cancer cells used for the TX-53 mission were grown without contact to the cell chamber (CC) material, with direct contact to the CC material, or in conditioned medium previously incubated with CC material (Fig. 1B). The examined medium did not show any pH changes due to the PC material. In addition, microscopic analysis of the cells presented comparable cell numbers and viability, with the cells keeping their characteristic morphology. To ensure cell viability in the PC environment, Annexin-V was investigated via flow cytometry. All three scenarios presented cell viabilities between 83% and 95%, while the number of apoptotic and necrotic cells was low (data not shown).

### Construction of the experiment container and their role in the sounding rocket mission

#### Cell chambers

**Figure 2:**
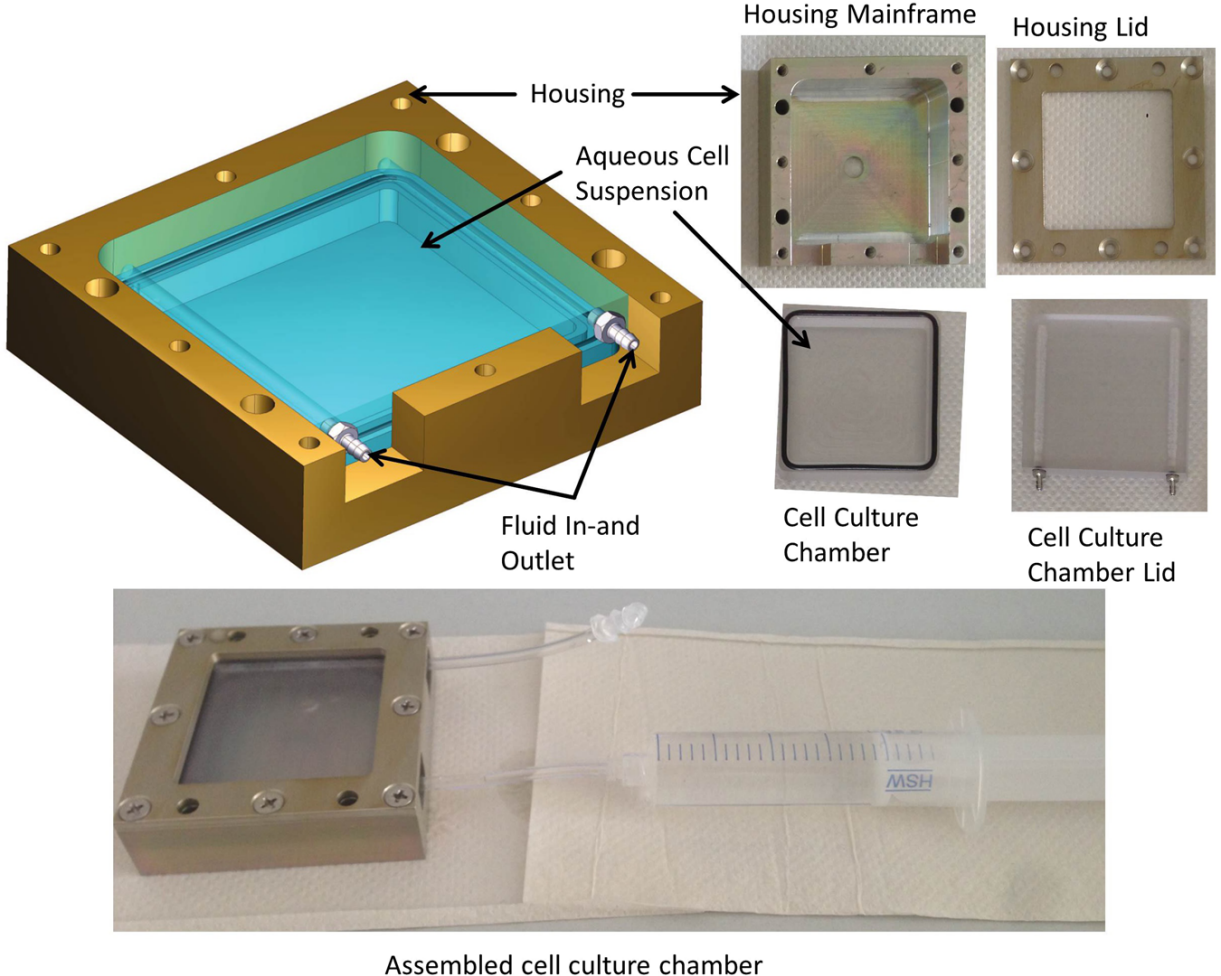
Components of the culture chamber (CC). The CC is composed of four parts. Cell culture chamber and lid are in direct contact to the cells. Housing and lid are used to keep the CC together.

The CCs provided a growth area allowing harvesting sufficient material for further analyses together with a significant number of replicates (Fig. 2). They are composed of two parts (Fig. 2). The cell holding chamber consists of two pieces milled from a PC block (blue). The lower part is 0.5 cm deep and has a cell growth area of 5 × 5 cm. The bottom was not special treated, as viability test and cell number determination presented good biocompatibility. The lid is also composed of PC. It holds two vertical tubes, which are studded with small holes pointing to the inner side of the cell chamber. The tubes are used for fluid in- and outlet. An O-ring embedded into the border of the lower chamber ensures proper sealing.

The PC culture chambers are embedded into an aluminum housing (gold). The housing lid presses the two PC pieces together when screwed on. It holds, in addition to the screw holes, four holes, which are used to connect the culture chambers to the fixation unit. All parts of the culture chamber are autoclavable and were disinfected prior to cell seeding.

#### Experimental Unit and Rigid platform

**Figure 3:**
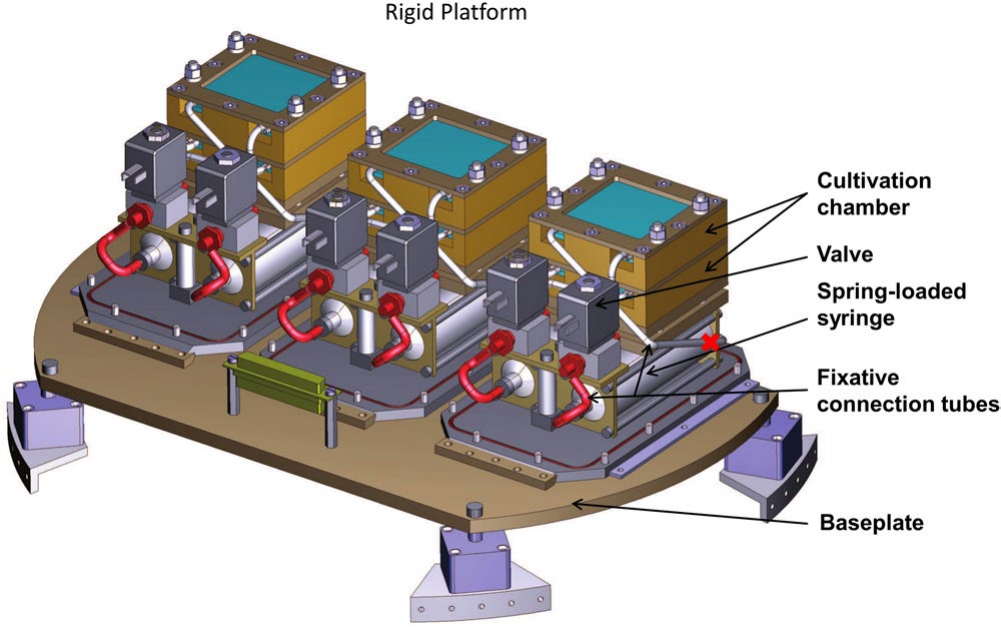
Rigid platform with three experimental units. One unit is composed of two CCs (gold) connected with a silicon tube (red) to two spring-loaded fixative syringes (light grey) which are triggered by two valves (dark grey) accordingly.

One experimental unit (EU) consists of two spring-loaded syringes and can hold two culture chambers (Fig. 3). The syringe is not only holding the fixative, but also is used as the reservoir for the medium, which leaves the cell cultivation chamber when triggered. The loaded syringes are connected to the culture chambers via silicon tubing. The triggers of the loaded system are valves, which squeeze the tubing to keep the pressure in the syringes. By releasing the squeeze on the tube, the spring pushes the fixative into the culture chamber, while the medium is guided through the outlet and into the back of the syringe. Syringes can be triggered separately, allowing fixation on various time points. Three experimental units are mounted to the rigid platform resulting in six cell culture chambers.

The EUs were prepared by Airbus Defence and Space. After assembling the parts and connecting the CCs, the EUs were introduced into a pressure resistant envelope and tested for pressure leakage in an evacuation chamber to exclude pressure-leakage.

#### Pivotal platform

**Figure 4:**
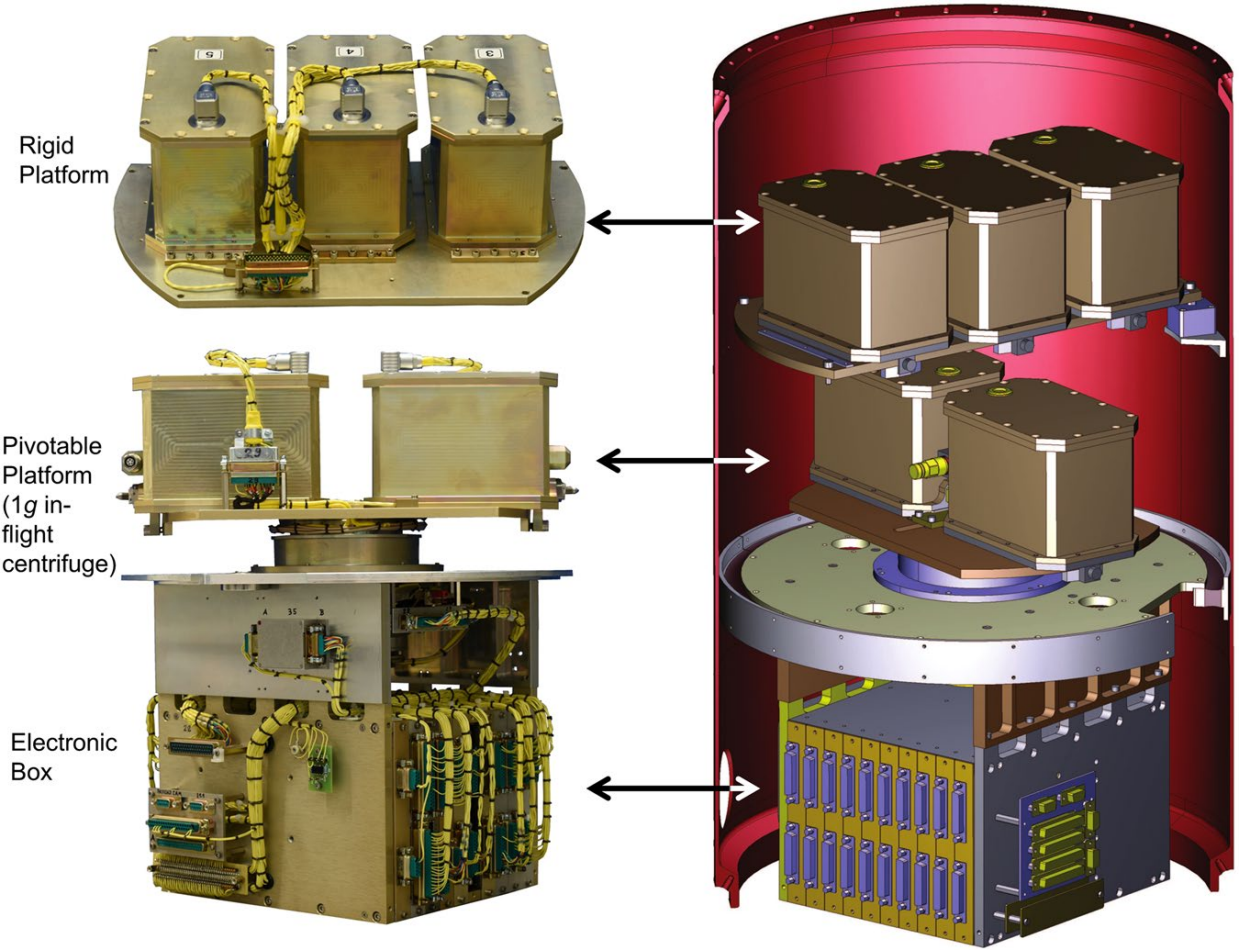
TEXUS 53 THYROID experiment. Left side: both platforms and electronic box. Right side: CAD drawing of integrated experiment module with external structure (red).

Experimental units, as described above, are also mounted on the pivotal platform (Fig. 4). The pivotal platform is connected to a motor, which enables centrifugation of samples at 1 *g* during rocket flight. The centrifuge is started immediately after entrance into microgravity, giving the researcher the opportunity to distinguish between accumulative effects of hyper-*g* during takeoff and 1 *g*, and hyper-*g* in addition to µ*g*. The centrifuge holds two experimental units, resulting in four samples. Due to the orientation of the cells on the centrifuge, they are exposed to hyper-*g* vertically while they feel 1 *g* horizontally (Supplemental Fig. 1). This increased the need for additional controls, referred to as 1 *g* in-flight simulation. In parallel to the rocket flight, experimental units on ground were either placed in a 90 degree tilted position to simulate the horizontal stimulation of the in-flight centrifuge or a non-tilted position as regular control for 6 minutes and were afterwards remote controlled chemically fixed.

### Impact of microgravity and hypergravity on gene expression

**Figure 5:**
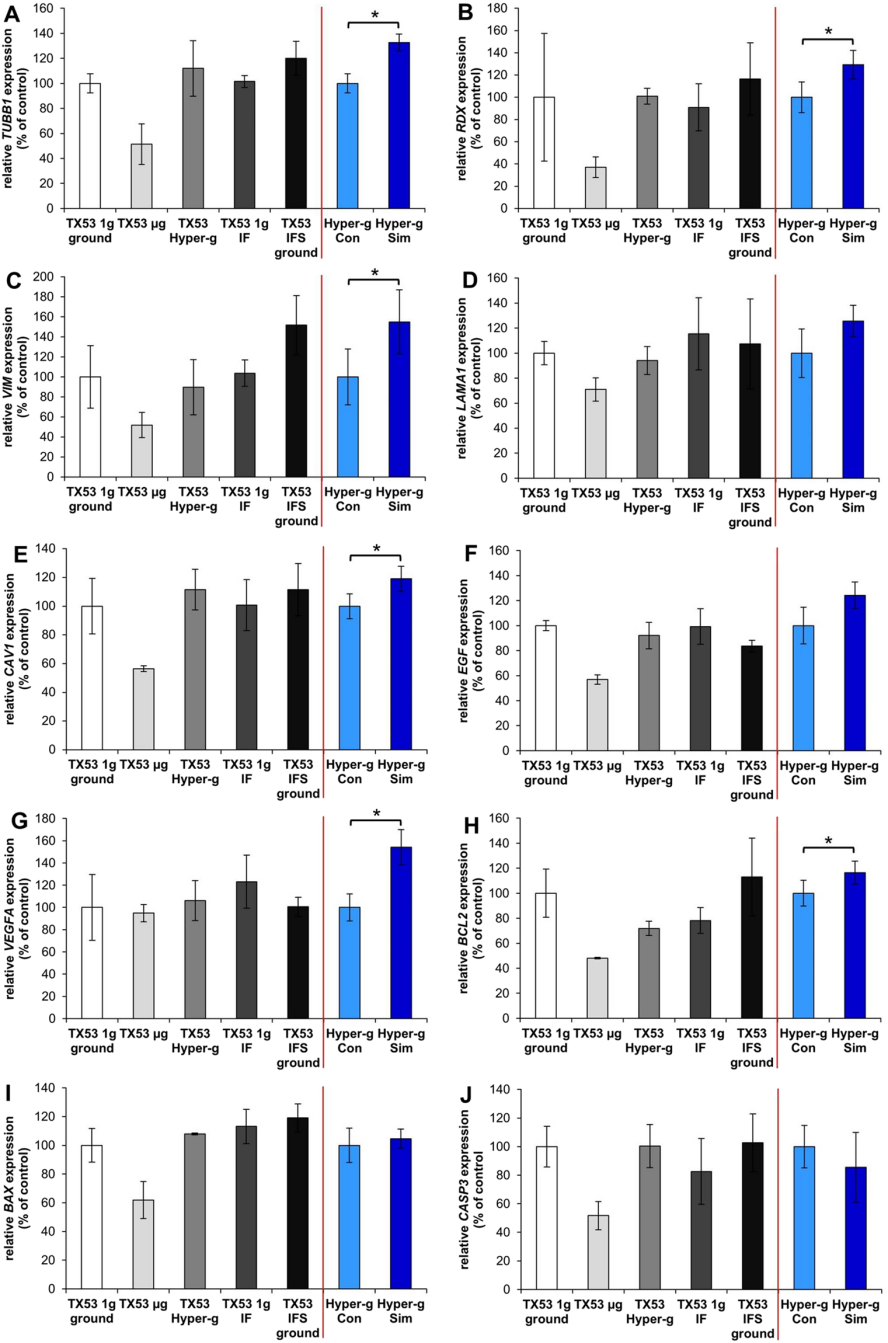
Gene expression of selected genes involved in several biological processes. (**A**) Beta-tubulin (*TUBB1*), (**B**) Radixin (*RDX*), (**C**) Vimentin (*VIM*), (**D**) Laminin alpha 1 (*LAMA1*), (**E**) Caveolin 1 (*CAV1*), (**F**) Epidermal growth factor (*EGF*), (**G**) Vascular endothelial growth factor (*VEGFA*), (**H**) Bcl-2 (*BCL2*), (**I**) Bax (*BAX*), (**J**) Caspase-3 (*CASP3*) and data not shown Beta-actin (*ACTB*), Ezrin (*EZR*), Moesin (*MSN*), Talin 1 (*TLN1*), Integrin beta 1 (*ITGB1*) were analysed after exposure to various conditions. TX53 1 *g* ground is the ground control during the mission (n = 3); TX53 µ*g* describes the sample which was exposed to the launch acceleration and the following µ*g*-phase (n = 2); TX53 Hyper-*g* describes the sample which was only effected by launch acceleration (n = 3); TX53 1 *g* IF describes the sample which was first exposed to the launch acceleration and then centrifuged at 1 *g* during the µ*g*-phase (n = 3); TX53 IFS ground presents the simulation of the inflight centrifuge as the samples were stimulated horizontally (n = 3); Hyper-*g* control (n = 5) and hyper-*g* simulation (n = 5) present control and simulation of worst case acceleration possible during a sounding rocket launch. All values are given as mean ± standard deviation. *p < 0.05.

In order to gain reliable information about alteration of gene expression patterns the experimental groups depicted in Fig. 1C were prepared for the sounding rocket experiment as described under Material and Methods. After the mission, gene array experiments were performed. In addition, qPCR analyses were done on target gene products which were differentially regulated in real microgravity during parabolic flight mission and space experiments^11,12,14,18^. To achieve a high comparability, we included the results of all possible scenarios described in the material section. These data are given in Fig. 5.

**Figure 6:**
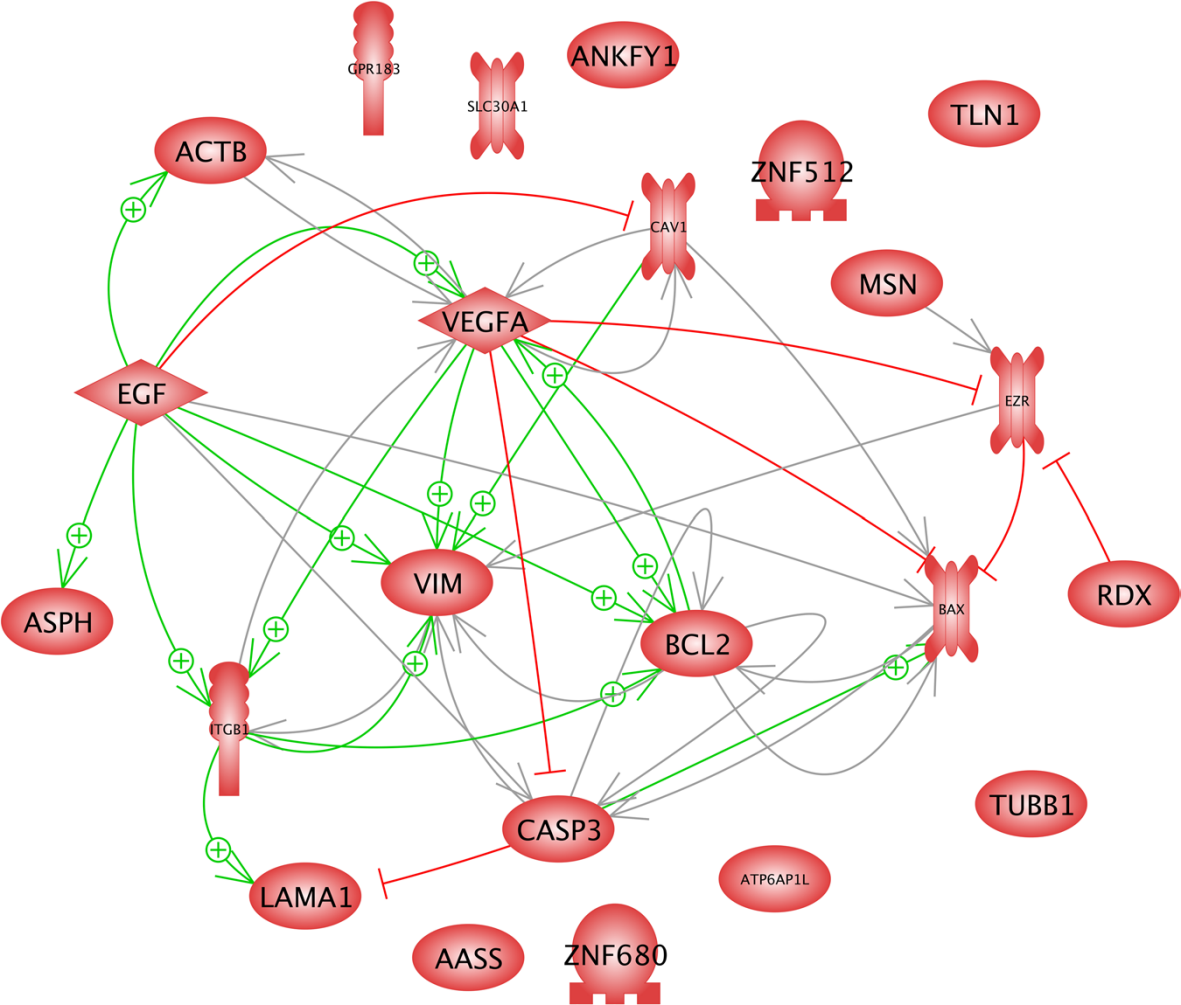
Interaction of investigated gene and gene products at gene level. Green arrows indicate up-regulation, red arrow mean down-regulation.

**Figure 7:**
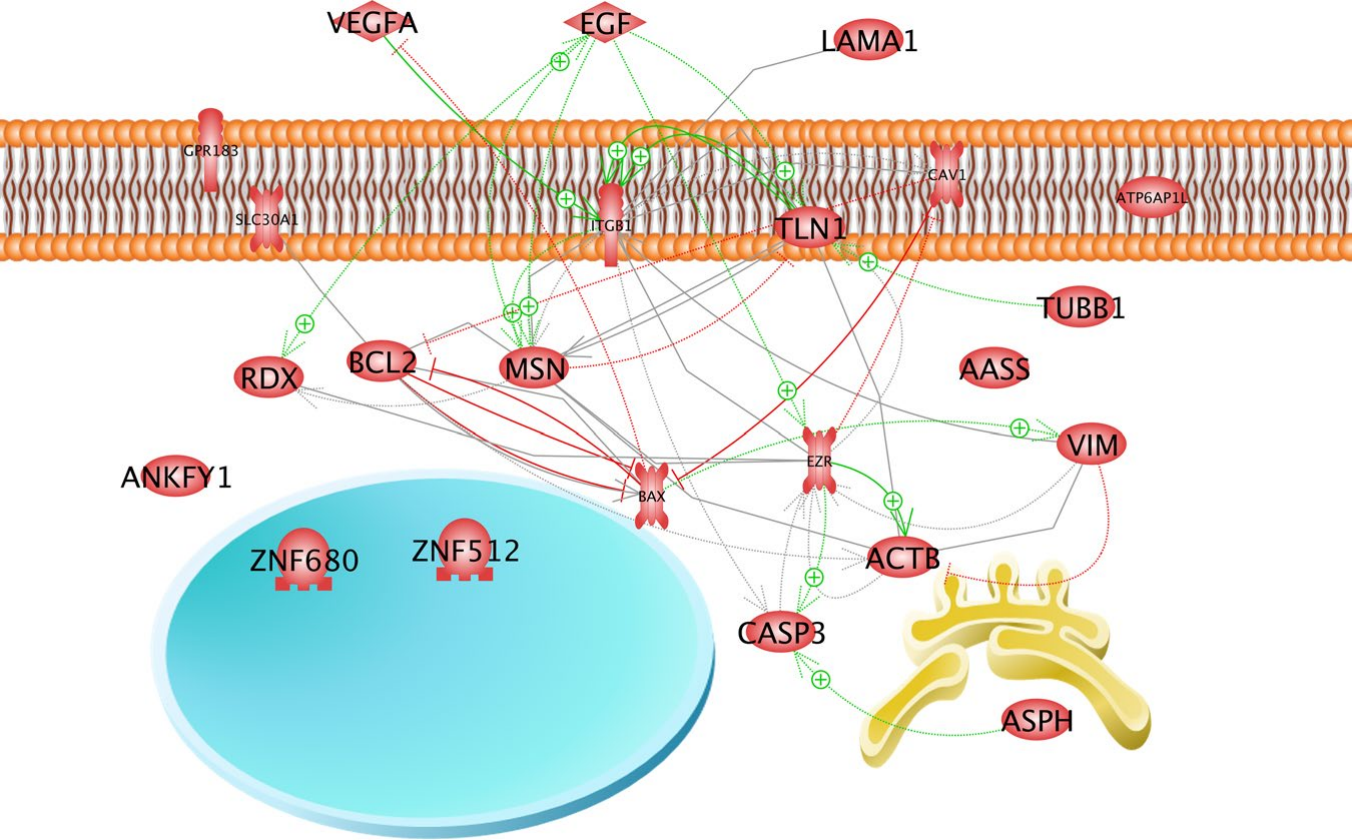
Interaction of investigated proteins and their localization within the cell. Blue circle indicates nucleus, yellow figure indicates ER, pink shows the membrane. Solid lines indicate binding, solid arrows indicate regulation by direct interaction, dashed arrows indicate indirect regulation via other cellular components.

We tested for expression changes in cytoskeletal and associated genes such as β-actin (*ACTB*), β-tubulin (*TUBB*), vimentin (*VIM*), ezrin (*EZR*), radixin (*RDX*), and moesin (*MSN*). In addition, the extracellular matrix gene laminin (*LAMA1*) and the membrane associated genes caveolin 1 (*CAV1*), talin 1 (*TLN1*) and integrin beta 1 (*ITGB1*) were investigated. Furthermore, the expression of the signaling cytokines epithelial growth factor (*EGF*) and vascular endothelial growth factor A (*VEGFA*) was determined. Finally, we focused on the apoptosis involved factors bcl2 (*BCL2*), bax (*BAX*) and caspase 3 (*CASP3*). Beside *TUBB1* all these genes form a network of interaction. These genes and their products are influencing each other at gene and protein levels (Figs 6,7). Central functions are exerted by the genes of the cytokines *EGF* and *VEGFA* (Fig. 6), which are usually secreted in the culture supernatant (Fig. 7). They have either enhancing (green arrows) or down-regulating (red lines) effects on the genes of the cytoskeletal proteins actin, vimentin, ezrin, and integrin beta 1, and of the scaffolding protein caveolin-1 as well as on those of the apoptotic factors CASP3, BAX and BCL-2. The cytoskeletal proteins and apoptotic factors investigated are located within the cells. They are affected by the cytokine EGF in an indirect manner (Fig. 7, dashed) via corresponding membrane inserted receptors. But amongst themselves, they may influence each other indirectly via additional proteins (Fig. 7, dashed lines) or directly by physical interaction (solid arrows) or even complex formation (solid lines) as shown for zinc transporter 1, BCL2, moesin and integrin-beta-1 (Fig. 7).

All scenarios introduced before were tested for expression of the mentioned factors. We first determined the expression changes during worst-case hyper-*g* scenario produced by a centrifuge for 1 min (referred to as Hyper-*g* Con and Hyper-*g* Sim). We found a significant up-regulation of *TUBB1*, *RDX*, *VIM*, *CAV1*, *VEGFA* and *BCL2* (Fig. 5A,B,C,E,G and H). *MSN* and *TLN1* (data not shown) as well as *LAMA1* and *EGF* (Fig. 5D,F) presented a non-significant up-regulation in this scenario while *ACTB*, *EZR*, *ITGB1* (data not shown) like *BAX* and *CASP3* (Fig. 5I,J) gene expression remained unchanged. These results reveal an up-regulating influence of VEGF-A on the *BCL2* gene (green arrow) and a down-regulating effect on BAX (red line) (Figs 6, 7). But unregulated BAX suggests that the effect of VEGF-A down-regulating the expression of this factor is balanced by an effect of EGF favoring BAX expression. In contrast, up-regulated *BCL2* seems to be due to a positive effect of EGF and VEGF-A. Both cytokines appear also to be involved in up-regulation of vimentin.

The control scenarios (referred to as TX53 1 *g* ground, TX53 1 *g* IF, TX53 IFS ground) and the hyper-*g* (referred to as TX53 Hyper-*g*) did not reveal significant variations. Even the gene array experiment on the cells of these scenarios did not reveal any gene that changed at least two-fold (Table 1). Only an ANOVA analysis over the gene array results on cells obtained from theses 4 scenarios suggests variation of eight genes (Table 1). None of the detected genes were identical to those selected for the experiments on the basis of earlier experiments. However, two of these genes fit within the interaction network of the well-known candidates for gravi-sensitivity among other (Figs 6, 7). The most significantly regulated gene was *ASPH* undergoing 5% FDR. It up-regulates *EGF* and its product aspartate beta-hydroxylase supports the action of CASP3 (Figs 6, 7).

**Table 1:** Cell number determination.

Container	1d		4d	
	Initial #	Final #	Initial #	Final #
T25	3 × 10^6^ (100%)	387500 (12.9%)	1.5 × 10^6^ (100%)	1.1 × 10^6^ (73.3%)
H/W	3 × 10^6^ (100%)	187500 (6.25%)	1.5 × 10^6^ (100%)	863000 (57.5%)
T25	2 × 10^6^ (100%)	1.6 × 10^6^ (80%)		
H/W	2 × 10^6^ (100%)	1,73 × 10^6^ (86.25%)		

Unfortunately, an issue happened during the installation of one of the pressure resistant envelopes which produced a kink in the fixative tubing (Fig. 3, indicated by a red cross) and prevented proper fixation. Due to the non-transparent cabin there was no possibility to check for any kinks in the tubing after installation and before takeoff. Therefore, the cells harvested from the µg samples did not allow a significant microarray gene analysis. qPCR experiments showed that the gene expression in µ*g*-samples is markedly, but not significantly down-regulated in most of the tested gene products (Fig. 5).

Only *VEGFA* remained clearly unregulated in µ*g* samples. Though it seems to lack an anti-apoptotic effect, as the tendency of regulation of *BCL2* seems to be opposite in µ*g* as compared to hyper-*g* (Fig. 5). Under the microgravity condition, the genes of the cytoskeletal proteins show though not significant but clear tendencies of down-regulation. Especially ezrin and radixin, which are both under the regulation of *EGF*, appear most clearly down-regulated.

## Discussion

Cell biology in real microgravity is currently a hot topic in space research^20^. To perform cell culture experiments in space a functioning, automated hardware is necessary. Several biological experiments with plants, mice or mammalian cells have been performed under microgravity conditions^11,13,19,21^. A few articles published the concept of experimental units for application on the ISS and in space^19,22–24^.

In this study, we describe the successful construction of a hardware container suitable to transport and culture human cells in real microgravity realized by the sounding rocket mission TEXUS 53 and the cell biological experiment THYROID. Using this hardware, we investigated the molecular biological changes of human follicular thyroid cancer cells (FTC-133 cell line) exposed to r-µ*g* and hyper-*g* during a sounding rocket mission. The aim of this project was to monitor changes in gene expression occurring during short-term µ*g* in thyroid cancer cells measured by microarray analysis and following quantitative rtPCR.

These studies might help to further understand the observed impact of µ*g* on biological processes and the formation of 3D aggregates of normal and malignant cells^6,8,25,26^, explaining the mechanisms underlying the µ*g*-induced changes in growth behavior, differentiation, proliferation, cell adhesion, migration as well as the increased apoptosis and alterations of the cytoskeleton and extracellular matrix proteins^25^.

In 2011, we investigated FTC-133 thyroid cancer cells during a parabolic flight campaign (PFC) and during the Sino-German Shenzhou-8/SIMBOX space mission^12^. A microarray analysis performed after the PFC delivered 63 moderately regulated transcripts after 22 s of r-µ*g*, while 2881 significantly regulated (>2 fold) transcripts were recognized after 10 days on the RPM or in space^12^. The latter results showed genes of various biological processes such as apoptosis, cytoskeleton, cell adhesion/extracellular matrix, angiogenesis and others differentially regulated under altered gravity conditions^12^. Interestingly, a microarray analysis performed after another PFC with experiments on thyroid cancer ML-1 cells revealed 102 significantly (>2 fold) down-regulated, and 46 up-regulated genes^27^, which also coded for proteins involved in biological processes mentioned above. In addition, they revealed sensitivity of *ASPH* and *EBI2* genes found also in this microarray analysis together with *ZNF512*, which had been noticed earlier in chondrocytes recovered from PFC^28^.

Here, we focused on selected genes also belonging to these processes mentioned above and investigated the low-differentiated FTC-133 follicular thyroid cancer cells exposed sounding rocket flight, which includes 1 min of extreme hyper-*g* and 6 min of µ*g*.

The cytoskeletal genes *ACTB*, *TUBB1* and *VIM* were down-regulated by µ*g*, whereas hyper-*g* during the flight did not alter the gene expression of these genes. In contrast, a centrifugation of the cells on Earth induced an up-regulation of the *VIM* and *TUBB1* mRNAs, indicating that factors like launch stress and vibration may impact the cytoskeleton.

The ERM protein group comprises the three proteins ezrin, radixin and moesin and is known to crosslink actin with the cellular plasma membranes regulating biological processes like reorganization of actin cytoskeleton, cell survival, membrane dynamics, cell adhesion and migration^29^. In a preceding set of experiments actin bundles changed rapidly when a sounding rocket entered the µg-phase and an accumulation of F-actin at the outer membranes of different cell types was detectable after parabolic flights (PF). Simultaneously *EZR* was down-regulated in cells expressing Lifeact-GFP, but up-regulated in normal FTC-133, while RDX and MSN remained un-changed in both types of cells^18^. In the present study, after 6 min of µ*g EZR*, *RDX* and *MSN* mRNAs were down-regulated compared to all other groups.

This may be caused by enhanced hyper-*g* during the launch phase as a similar increase of *EZR*, *MNS*, and *RDX* is observed under 1.8 *g* conditions^18^. Of the three ERM genes, *EZR* was most strikingly down-regulated. This gene change resembles the result obtained after PF of endothelial cells, where *EZR* was down-regulated only, but *MSN* and *RDX* remained unchanged^30^. The relationship was turned around in 7-day simulated (s)-µ*g* cultures, when the *EZR* gene was mostly up-regulated^31^.

Interestingly, we detected a slight reduction of the *LAMA1* gene expression in the µ*g* group. This cell adhesion molecule and extracellular matrix protein is known to be altered when different cell types are exposed to s-µ*g* conditions^8,9,18,32,33^. A reduction was reported in adult retinal pigment epithelium cells and increases in dental pulp stem cells^32,33^. During the PFC FTC-133 cells exhibited no change in *LAMA1* gene expression after 31 parabolas^18^. Laminins are important for cell adhesion, the shape of the cells, phenotype, their differentiation, and movement.

The *CAV1* mRNA expression was reduced in microgravity samples. CAV-1 is a known member of several pathways such as caveolar-mediated endocytosis signaling, endothelial nitric oxide synthase (eNOS) signaling, integrin signaling, and others. Its role in spheroid formation is not clear. The *CAV1* mRNA was elevated in thyroid ML-1 cells and in chondrocytes after a PF^27,34^ as well as in 24-hour mechanical-unloading-exposed human umbilical venous endothelial cells, which was associated with a decrease in the length and width of the cells^35^. After a 5- and 7-day cultivation of EA.hy926 cells, this gene was down-regulated^31^. We recently demonstrated that caveolin protein is reduced in multicellular spheroids and elevated in adherent cells^36^, indicating that caveolin is a key regulator for growth and spheroid formation.

Talin (*TLN1*) is normally detectable in large amounts in focal adhesions and links integrins to the actin cytoskeleton^37^. Talin interacts directly with β-integrin (Fig. 7)^38^, therefore we analyzed the gene expression of *TLN1*. The *TLN1* mRNA expression was reduced in microgravity samples and unaltered in all other groups. It is known that TLN1 is involved in RPM-dependent thyroid cancer spheroid formation^15^. Regarding the findings described above, the *TLN1* gene expression might point to the time frame in which the detachment and subsequent formation of MCS occurs.

In addition, we observed a reduced amount of epidermal growth factor (*EGF*) mRNA in µ*g-* samples. In a spaceflight experiment, EGF has shown to be involved in the scaffold-free formation of three-dimensional (3D) aggregates by FTC-133 cells with altered expression of *EGF* and *CTGF* genes under r- µ*g*^11^. The multicellular spheroids contained increased amounts of *EGF* mRNA compared to control cells.

Another growth factor known to be important for 3D growth is Vascular Endothelial Growth Factor (VEGF) which had demonstrated an influence on the ITGB1-LAMA1-TLN1 complex (Fig. 7, solid grey lines)^39,40^. VEGF was only up-regulated by centrifugation compared to control cells and was not altered during the rocket flight. In endothelial cells VEGF was unregulated after a PF^31^. But in chondrocytes VEGF-A was enhanced after simulation of the hyper-g phases of a PF^28^. After 10 days in space a down-regulation of *VEGFA* was detectable in adherent cells and spheroids^12^. These data were confirmed by a RPM experiment. In contrast, data obtained from a parabolic flight mission revealed no change in the gene expression of VEGF-A^12^, which support the results of this study. From these findings, it is obvious that its gene expression is altered at later time points by microgravity suggesting a role for VEGF-A in growth and proliferation of the spheroids.

It is known from earlier studies that apoptosis is involved in cell detachment and formation of spheroids^10,15^, therefore we investigated *BCL2*, *BAX* and *CASP3* mRNAs. *BAX* and *BCL2* genes were down-regulated in the µ*g* group.

The gene array experiments revealed eight genes that changed at least two-fold, none of the detected genes were identical to those selected for the experiments on the basis of earlier experiments. However, two of these genes fit within the interaction network of the well-known candidates for gravi-sensitivity.

Taken together, this sounding rocket flight carrying a cell biological experiment in an automatic functioning hardware has demonstrated its suitability for realizing cell biology studies under microgravity conditions even though some minor adaptations have to be introduced.

Moreover, the data show that the expression of key genes observed during earlier experiments at various time points is altered after six minutes of r-µ*g*. Hence, it further expands our knowledge of time-dependence of response of these genes to µ*g*. *EZR* seems to be fast responding to changes of gravity. While the *CAV-1* gene is up-regulated in a very early phase and down-regulated afterwards, gravity-dependent reactions of the *VEGFA* gene begin later starting with a transient up-regulation followed by down-regulation.

Future studies should be performed to investigate phosphorylation of proteins and morphological changes of the cells with the help of a laser scanning microscope suitable for space experiments.

## Conclusions

The results of all completed experiments enabled the construction of a reliable container for thyroid cell culture during a sounding rocket mission, which flew successfully in January 2016.

The developed automatic cell culturing system was demonstrated to be suitable for sounding rocket experiments with human adherent cells and can be used for future cell biological experiments by space researchers.

But the hardware needs improvement by guiding the tubing through an aluminum tube at critical positions in order to avoid future adversities. These cell culture experiments in real microgravity are necessary to increase our knowledge about the alterations of cells exposed to microgravity. This information is important to protect our health because in the near future, many humans will travel in space and missions are planned to the Moon and Mars.

## Material and Methods

### Cell cultures

The human thyroid cancer cell line FTC-133 is well-established and was flown in space several times prior to this sounding rocket flight (Fig. 1A)^11,13^. The cell line is derived from a lymph node metastasis of a follicular thyroid carcinoma. Even though the cell line retained some thyrocyte functions like responsiveness to thyrotropin, a number of chromosomal changes and p53 mutations were detected^41^.

The cells were cultured in RPMI 1640 medium (Life Technologies) supplemented with 10% FBS and 100 U/mL penicillin and 100 μg/mL streptomycin (Sigma). Cultivation took place in a humidified incubator at 37 °C and 5% CO_2_.

### Biocompatibility tests

The hardware used was designed and constructed by Airbus Defence and Space. In order to prove the biocompatibility of the CCs, FTC-133 cells were seeded into vented T25 cm^2^ cell culture flasks at a density of 10^6^ cells per flask, giving them a minimum of 24 hours to adhere.

To test the biocompatibility of the cell chamber material, three scenarios were applied:

In scenario S1 the cells were harvested and analyzed after four days of cultivation without contact to other materials (control samples; Fig. 1B(A)).

In S2 the polycarbonate (PC) material of the culture chamber (CC) was placed into the cell culture flasks to assure direct contact between the cells and the material. After exposure for four days the cells were harvested and analyzed (Fig. 1B(B)). Finally, in S3 the FTC-133 cells were incubated for 4 days with conditioned medium, previously exposed to the culturing chambers’ PC material for six days (Fig. 1B(C)).

After these tests, the apoptotic status of the cells was assessed using the Annexin V-FITC Apoptosis Detection Kit I (BD Bioscience) as previously described^11,19,42^.

The stained cells were analyzed using a FACSCanto II (Becton Dickinson), data analysis was attained by Flowing Software (Version 2.5.1). Each measurement was composed of a count of 6 × 5000 cells. To visually examine cell viability, cells were observed and photographed using an Axiovert 25 Microscope (Carl Zeiss Microscopy, LLC, USA) and a Canon EOS 550D camera (Canon GmbH, Krefeld, Germany).

### Determination of the appropriate cell number

To test which cell number had to be seeded per cell culture flask to result in an appropriate amount of RNA for downstream analyzes, the optimal cell density was determined. As sounding rocket launches are restricted to suitable weather conditions and postponements are common, we also included the possibility of delays. Table 1 summarizes the different tests. The cells were seeded with a defined cell density into the CCs with growth areas of 25 cm^2^ or into T25 cm^2^ flasks and incubated for either one or four days. After reaching the time point of interest, the cells were harvested by trypsinization, counted by Neubauer technique and stained with trypan blue to determine the cell viability.

### Experimental setup

To allow for statistical analysis, the experiments shown in Fig. 1 were carried out in triplicates: three samples were fixed with RNA*later* (Sigma) one min after launch (referred to as TX53 hyper-*g*). Three samples were located on the in-flight centrifuge and were fixed prior to reentry of the rocket (referred to as TX53 1 *g* in-flight) and finally, four samples should have been fixed after the microgravity (µ*g*) phase. However, only two samples were triggered. In addition to the flight samples, ground controls and hypergravity (hyper-*g*) simulations were conducted. Two different ground controls must be taken into account, which resulted from the positioning of the cells on the in-flight centrifuge. During the flight the cells were stimulated vertically at 1 *g* on a centrifuge. To simulate this stimulation, the hardware was positioned at a 90 degree angle. Controls were performed in parallel to the rocket flight, in ground-based hardware with an automated fixation unit (Fig. 1C).

### Cell cultures prior to launch at ESRANGE

**Figure 8:**
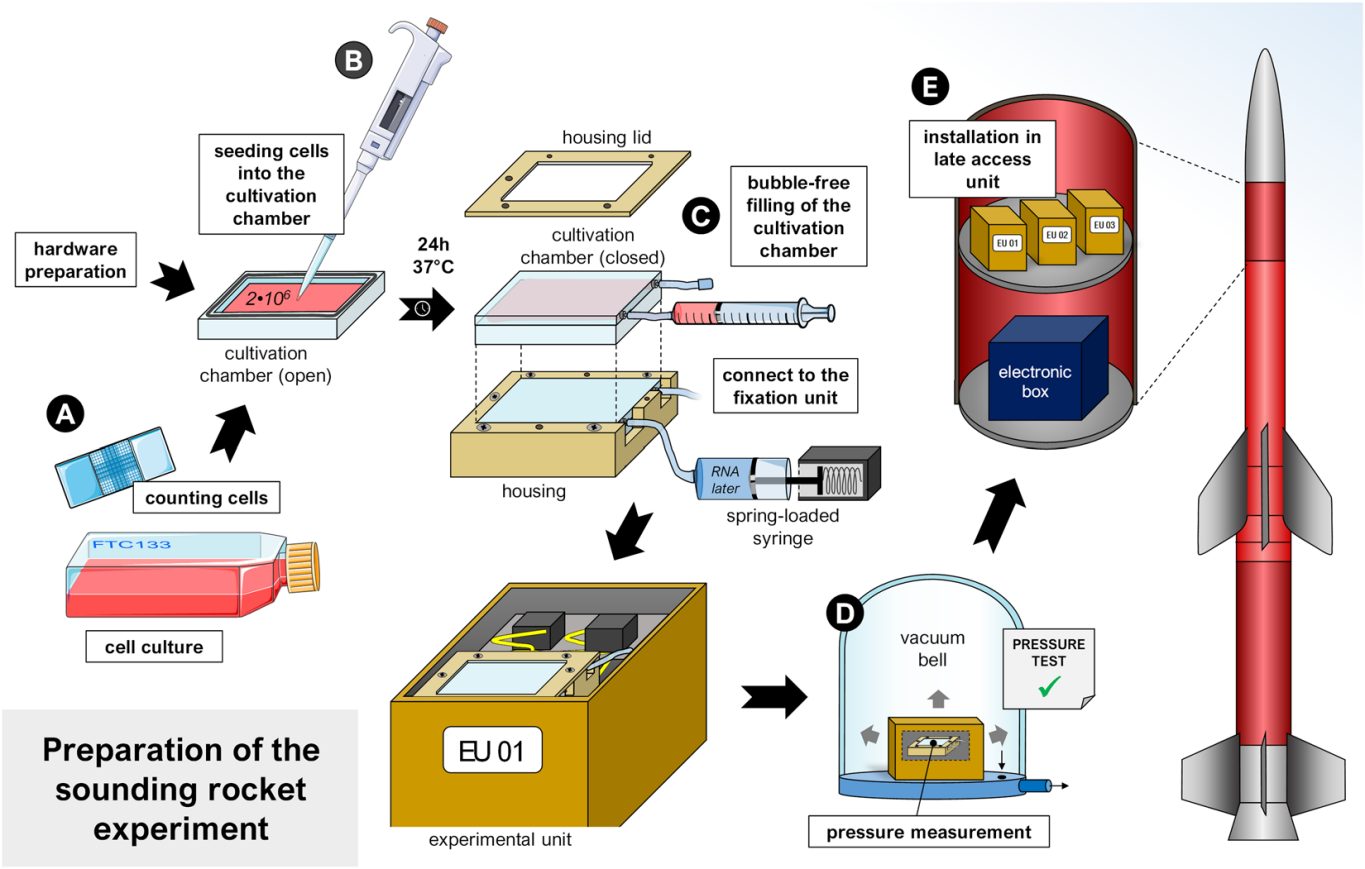
Preparation of the sounding rocket experiment at the laboratory of the Swedish Space Corporation (SSC) at the Esrange launch center, Kiruna, Sweden. (**A**) cell culture: (**B**) cell seeding with defined cell number in cultivation chamber; (**C**) bubble-free filling of the cultivation chamber; (**D**) installed chamber in the experimental unit and pressure measurement; (**E**) installation in late access unit. Parts of the figure were drawn by using pictures from Servier Medical Art. Servier Medical Art by Servier is licensed under a Creative Commons Attribution 3.0 Unported License (https://creativecommons.org/licenses/by/3.0/).

The FTC-133 cells were set up one week before launch (Fig. 8A). As sounding rocket launches are restricted to suitable weather conditions and postponements are common, we also included the possibility of delays and repeated the setup procedure during the following days.

One day prior to the scheduled rocket launch, the cells were seeded into the CCs (Fig. 8B). Therefore, adherent FTC-133 cells were harvested from culture flasks via trypsinization and counted via the Neubauer technique (Fig. 8A). As a result to the previous determination of the optimal cell number, 2 × 10^6^ cells per CC were directly seeded in 6 mL medium (Fig. 8B). CCs were stored inside their housing in a 37 °C incubator overnight to allow cell adhesion.

On launch day, the culture medium was exchanged and the chambers were filled entirely, bubble-free (Fig. 8C). The flasks were connected to spring-loaded syringes, which were equipped with a solenoid valve and filled with RNA*later*, and subsequently installed in the experimental units (EUs). A pressure test was performed following assembly: The EUs were placed in a vacuum bell with low pressure while sensors logged the inside pressure of the EUs, which should not change during this procedure (Fig. 8D). Upon passing this test, the EUs were installed on the rigid platforms of the rocket as late access units (Fig. 8E). Then the EUs were incubated at 37 °C until handover to Airbus Defence and Space.

### Sounding rocket flight

A sounding rocket flight is composed of different stressors^18^. During launch the rocket reached a maximum linear acceleration of 12.1 *g* (Fig. 1A, Supplemental Table 1). In addition to the acceleration, the rocket is spin stabilized during ascent, which also influences the cell chamber in case bubbles are present. T + 1 min after launch, the first and the second stage of the rocket motors are separated from the payload and the payload rotation is stopped via a yo-yo de-spin mechanism. This is followed by a nearly six-minute-phase of microgravity during which the payload describes a high parabola and reaches an apogee of approx. 260 km. Shortly after re-entry into Earth atmosphere, a parachute system is released to de-accelerate the payload and let it glide safely back to the ground. Typically, 90 minutes after landing, the payload is recovered which allows fast retrieval of the samples. Because the flight is rather short, all procedures on board are automatically executed. Supplemental Table 1 shows all data gathered during the flight.

### Hyper-g simulation

Cells were handled similar to the cell samples prior to launch. FTC-133 cells were seeded into T25 cm^2^ cell culture flasks at a density of 2 × 10^6^ cells and given 24 hours to adhere to the bottom of the flasks. The next day the samples were run at 18 × *g* for 1 min at 31 °C in a Heraeus Multifuge 3SR + (Thermo Scientific), in microtiter plate buckets (127 × 85 mm) to simulate the worst-case scenario during rocket launch acceleration. After each run, cells were fixed with RNA*later* for downstream analysis. 4 cell culture flasks were centrifuged at combined for a total of 4 times. Controls were placed in the same centrifuge for 1 min without centrifugation.

### Post-flight cell harvest

After the rocket flight, the cell samples were received fixed in RNA*later*. To harvest the cell material for post-flight analyses, the supernatant was removed and 1 mL of fresh RNA*later* was added. Using a small cell scraper, the cells were detached and brought into solution. The solution was transferred into 2 mL tubes. The cultivation area was washed twice with 1 mL RNA*later*, the solution transferred into the corresponding tubes. The cell suspension was kept at 4 °C until further analysis.

### Quantitative real time PCR

The RNA extraction was performed using the RNAeasy mini kit (Qiagen GmbH, Hilden, Germany) according to the manufacturer’s protocol. Concentrations were determined via Nanodrop 2000 (Thermofisher Scientific), as described previously^12,18^.

Complementary DNA was produced using the First Strand cDNA Synthesis Kit (Thermo Fisher Scientific) following manufacturer’s instructions. qrtPCR was performed using the SYBR^®^ Select Master Mix (Applied Biosystems, Darmstadt, Germany) and the 7500 Fast Real-Time PCR System (Applied Biosystems) to determine the expression levels of target genes, shown in Table 2. Selective primers were designed using PubMed primer blast, with a focus to span exon-exon boundaries where applicable, and on a T_m_ of 60 °C. The designed primers were synthesized by TIB Molbiol (Berlin, Germany). Samples were measured in triplicates and normalized to the 18 S rRNA as housekeeping control. The comparative threshold cycle (ΔΔCT) method was used for relative quantification of transcription levels, with 1 *g* set as 100%.

**Table 2:** Primers used for quantitative real-time PCR.

Factor	Primer name	Sequence 5′-3′
*18 S*	*18S-F*	GGAGCCTGCGGCTTAATTT
	*18S-R*	CAACTAAGAACGGCCATGCA
Actin Beta	*ACTB-F*	TGCCGACAGGATGCAGAAG
	*ACTB-R*	GCCGATCCACACGGAGTACT
Apoptosis Regulator BAX; *BAX*	*BAX-F*	GTCAGCTGCCACTCGGAAA
	*BAX-R*	AGTAACATGGAGCTGCAGAGGAT
Apoptosis Regulator BCL-2; *BCL2*	*BCL2-F*	TCAGAGACAGCCAGGAGAAATCA
	*BCL2-R*	CCTGTGGATGACTGAGTACCTGAA
Caspase 3	*CASP3-F*	CTCCAACATCGACTGTGAGAAGTT
	*CASP3-R*	GCGCCAGCTCCAGCAA
Caveolin-1	*Cav1-F*	CCTCCTCACAGTTTTCATCCA
	*Cav1-R*	TGTAGATGTTGCCCTGTTCC
Endothelial Growth Factor	*EGF-F*	TGCCAGCTGCACAAATACAGA
	*EGF-R*	TCTTACGGAATAGTGGTGGTCATC
Ezrin	*EZR-F*	GCAATCCAGCCAAATACAACTG
	*EZR-R*	CCACATAGTGGAGGCCAAAGTAC
Laminin Alpha 1	*LAMA1-F*	TGACTGACCTGGGTTCAGGA
	*LAMA1-R*	TGCTAGCACTCCTTGCTTCC
Moesin	*MSN-F*	GAAATTTGTCATCAAGCCCATTG
	*MSN-R*	CCATGCACAAGGCCAAGAT
Radixin	*RDX-F*	GAAAATGCCGAAACCAATCAA
	*RDX-R*	GTATTGGGCTGAATGGCAAATT
Integrin Beta 1	*ITGB1-F*	GAAAACAGCGCATATCTGGAAATT
	*ITGB1-R*	CAGCCAATCAGTGATCCACAA
Talin-1	*TLN1-F*	GATGGCTATTACTCAGTACAGACAACTGA
	*TLN1-R*	CATAGTAGACTCCTCATCTCCTTCCA
Tubulin Beta 1	*TUBB1-F*	CTGGACCGCATCTCTGTGTACTAC
	*TUBB1-R*	GACCTGAGCGAACAGAGTCCAT
Vascular Endothelial Growth Factor A	*VEGFA-F*	GCGCTGATAGACATCCATGAAC
	*VEGFA-R*	CTACCTCCACCATGCCAAGTG
Vimentin	*VIM-F*	TTCAGAGAGAGGAAGCCGAAAAC
	*VIM-R*	AGATTCCACTTTGCGTTCAAGGT

All sequences are given in the 5′-3′direction.

### Microarray analysis

The same RNA samples as used for the qPCR were examined via microarray analysis. Samples were analyzed via the Illumina HumanWG-6_V2_0_R3 array, which was normalized using the BeadStudio Gene Expression Module v3.3.7, in addition to quantile normalization without background correction. After the quantile normalization and exclusion of low or not expressed genes (minimum Illumina detection p-value > 0.05) a parametric ANOVA was applied to compare the conditions 1*g*-on-ground, 1*g*-in-flight, 1*g*-on-ground-in-flight simulation and hyper-*g*-inflight. µ*g* samples were not included into the measurement as a sample number of 2 is statistically irrelevant. Probes which undergo 5% FDR (Reference) are usually selected as differential expressed, however, only one of the measured genes, the aspartate beta-hydroxylase (ASPH) reached this condition (Table 3).

**Table 3:** Genes differentially expressed at a significance level of P < 10^−4^ in the ANOVA over the four conditions: 1*g*-on-ground, 1*g*-in-flight, 1*g*-on-ground-in-flight simulation and hyper-*g*-inflight.

SYMBOL	P	Q	Log2 expression (standard deviation)			
			1 *g* in flight centrifuge	1 *g* on ground	1 *g* on ground infl. sim.	hyper-*g* in flight
ASPH	6.25E-07	**0.017**	7.30 (0.016)	7.16 (0.020)	7.33 (0.009)	7.45 (0.026)
ZNF512	1.50E-05	0.125	8.31 (0.011)	8.61 (0.042)	8.52 (0.036)	8.28 (0.053)
ATP6AP1L	1.54E-05	0.125	7.02 (0.022)	7.09 (0.028)	7.01 (0.006)	6.88 (0.021)
ZNF680	1.80E-05	0.125	7.71 (0.014)	7.64 (0.038)	7.64 (0.018)	7.83 (0.007)
EBI2	4.53E-05	0.243	8.53 (0.076)	8.26 (0.109)	8.44 (0.056)	8.91 (0.057)
SLC30A1	6.14E-05	0.243	8.14 (0.045)	8.10 (0.099)	8.17 (0.084)	8.62 (0.040)
AASS	7.44E-05	0.243	7.25 (0.033)	7.31 (0.013)	7.31 (0.013)	7.16 (0.017)
ANKFY1	9.87E-05	0.243	7.01 (0.016)	6.87 (0.026)	6.94 (0.010)	6.88 (0.028)

Significances are printed in bold.

### Pathway analysis

To investigate mutual regulation of genes and to visualize localization and interactions between proteins, we entered the relevant UniProtKB entry numbers in the Pathway Studio v.11 software (Elsevier Research Solutions, Amsterdam, The Netherlands). Graphs were generated for gene expression and protein regulation and binding. The method was described previously^36,43^.

### Statistical evaluation

Statistical evaluation was performed using SPSS 15.0 (SPSS, Inc., Chicago, IL, USA). The Mann-Whitney-U-Test was used to compare 1 *g* and μ*g* conditions, as well as 1 *g* and hyper-*g* conditions. All data is presented as mean ± standard deviation (SD) with a significance level of *p < 0.05.

## Electronic supplementary material

Supplementary Information
