# Supplementary material for: Thyroid cancer cells in space during the TEXUS-53 sounding rocket mission – The THYROID Project
Source: Sci Rep. 2018 Jul 9;8:10355. doi: 10.1038/s41598-018-28695-1 (PMC6037793; doi:10.1038/s41598-018-28695-1)
Supplement: Supplementary file 1 — Supplementary Information [file 41598_2018_28695_MOESM1_ESM.pdf]

Thyroid cancer cells in space during the TEXUS-53 sounding rocket mission – The THYROID Project

Sascha Kopp[a#], Marcus Krüger[a#], Stefan Feldmann[b], Hergen Oltmann[b], Andreas Schütte[b], Burkhard Schmitz[b], Johann Bauer[c], Herbert Schulz[d], Kathrin Saar[e], Norbert Huebner[e], Markus Wehland[a], Mohamed Zakaria Nassef[a], Daniela Melnik[a], Stefan Meltendorf[f], Manfred Infanger[a], Daniela Grimm[a,g,h*]

[a] Clinic for Plastic, Aesthetic and Hand Surgery, Otto-von-Guericke-University Magdeburg, Leipziger Str. 44, D-39120 Magdeburg, Germany

[b] Airbus Defence and Space GmbH, Airbus-Allee 1, D-28199 Bremen, Germany

[c] Max-Planck Institute of Biochemistry, D-82152 Martinsried, Germany

[d] Cologne Center for Genomics, University of Cologne, D-50931 Cologne, Germany

[e] Max-Delbrück-Center for Molecular Medicine, D-13092 Berlin-Buch, Germany

[f] Experimental Pediatrics and Neonatology, University Hospital, Otto-von-Guericke University Magdeburg, Leipziger Str. 44, D-39120 Magdeburg, Germany

[g] Department of Biomedicine, Aarhus University, Wilhelm Meyers Allé 4, DK-8000 Aarhus C, Denmark

[h] Gravitational Biology and Translational Regenerative Medicine, Faculty of Medicine and Mechanical Engineering, Otto-von-Guericke-University Magdeburg, D-39120 Magdeburg, Germany

# both contributed equally to this work

Supplementary Information Table 1 and Figure 1

Supplemental Table 1: Flight data of TEXUS-53

OHB QUICKLOOK TEXUS – 53

Launching date	**January, 23**	2016
Launching time	**09:30:00**	LT
μ-g Time	**367**	Sec
Payload weight	**400.1**	Kg
Payload length	**5215**	Mm

Motor First stage

Peak thrust acceleration	8.3g @ 2.2	Sec
Mean thrust acceleration	5.2	G
Burnout	11.9	Sec
Motor Separation	12.9	Sec

Motor Second stage

Ignition	15.0	Sec
Peak thrust acceleration	12.1g @ 34.9	Sec
Mean thrust acceleration	6.6	G
Burnout 0%	43.4	Sec
Spin at burnout (derived from rate sensor signal)	2.8	Hz
YoYo despin	56.0	Sec
Roll rate after YoYo despin	-18.7 @ 57.6	°/sec
Motor separation	59.0	Sec

RCS Acquisition & Control

RCS enable		59.0	Sec
Acquisition phase		59.0 – 66.0	Sec
Manoeuvres at high thrust:			Sec
Manoeuvres at low thrust:	+Y	399.6 – 403.8	Sec
RCS low thrust enable		60.9	Sec
Lateral Destabilization and Spin-up		458.3	Sec
Achieved spin rate		151°/sec @ 476.3	Sec

Zero-g phase

μ-g achieved signal	66.0	Sec

Start of zero-g (< 1* 10exp-4 at sensors)	73.0	Sec
Time of apogee (derived from GPS data)	257.8	Sec
Apogee (derived from GPS data)	252.6	Km
End of zero-g (> 1* 10exp-4 at sensors)	440.0	Sec

Reentry & Recovery

Tip ejection	55.3	Sec
Reentry decelerations X= -15.4g Y= +9.7g Z=-0.2g	480.7/479.7/482.0	Sec
Maximum residual reentry deceleration	15.7g @ 481.0	Sec
Heat shield release	4.3g @ 583.9	Sec
Pilot parachute de-reefed	3.8g @ 593.5	Sec
Main parachute release	608.5	Sec
Main parachute de-reefed	2.1g @ 620.1	Sec
Sink rate	7.8	m/s

Housekeeping

Maximal skin temperature descent (POS4)	123.5°C @ 493.4	Sec

General

Loss of TM-Data & GPS last message at	877.0	Sec
Loss of GPS (4 samples) 0.0 – 3.0		Sec
Last coordinates derived from onboard GPS via TM	68°30.1385’	N
	21°02.6830’	E
Landing coordinates from onboard recorder	68°30.1589’	N
	21°02.6645’	E
Slant Range Distance (Azimuth = 357.9°)	67.9	Km
Landing Altitude	640	M
Impact decelerations (max.) X= -0.2g / Y= -0.3g / Z= 8.5g	887.6/887.9/887.6	Sec
Payload recovery time	5:30	H
Weather conditions	Fog in lower altitude, above clear sky	-23.5 °C
Air pressure (Launcher)	975	Mbar
Number of count-downs	1	

Supplementary Information

Supplemental Figure 1: Illustration of the different acceleration stimulations during launch and microgravity phase. In the hyper-*g* phase after lift-off the acceleration forces act perpendicularly towards the cells. When entering the microgravity phase the in-flight centrifuge turns on. Due to the special arrangement of the experimental units (brown boxes) on the pivotal platform, the cells are stimulated with 1*g* parallel to the cell growth surface. The figure was drawn from scratch by MK.

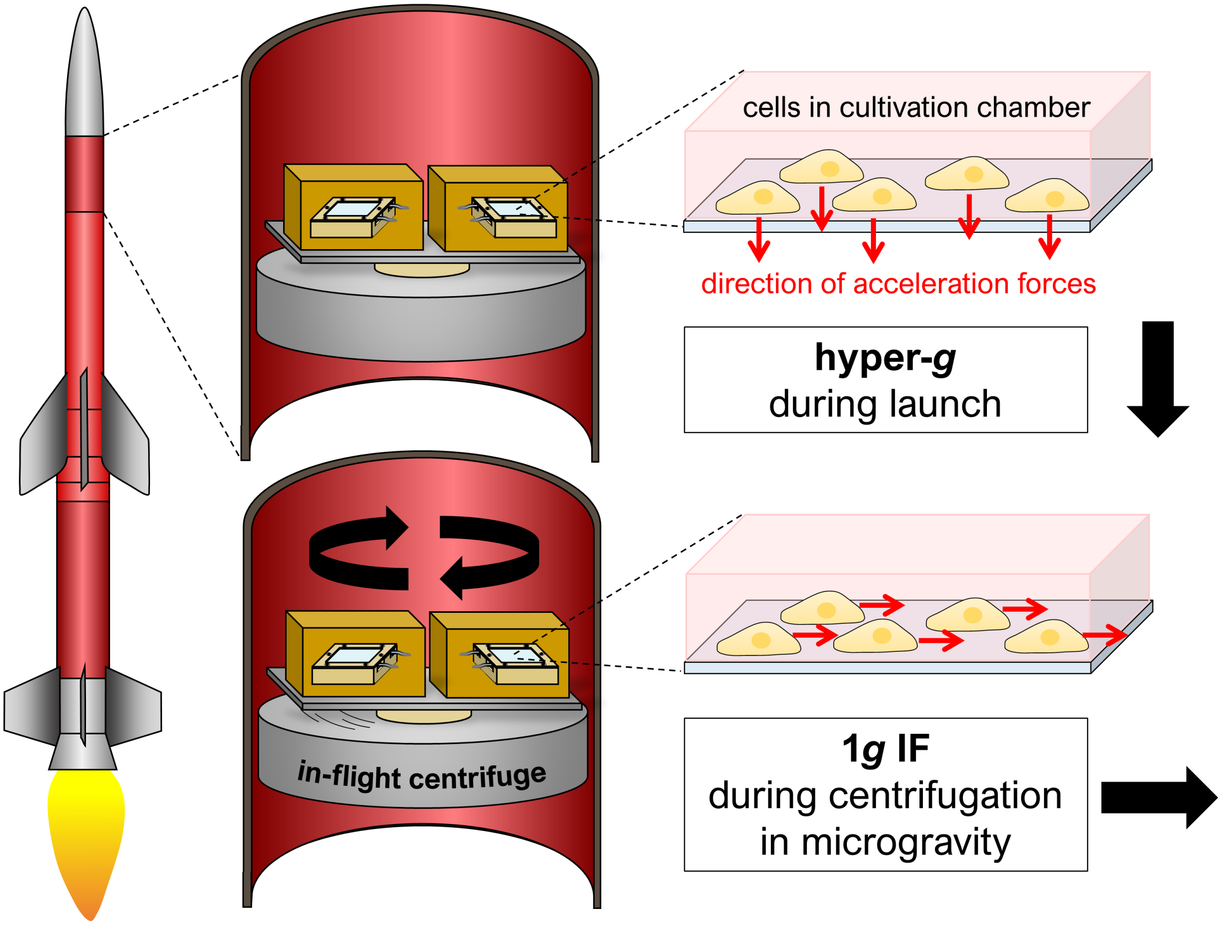
cells in cultivation chamber
direction of acceleration forces
hyper-g
during launch
in-flight centrifuge
1g IF
during centrifugation
in microgravity
